# Unequal Exposure to Occupational Stress across the Life Course: The Intersection of Race/Ethnicity and Gender

**DOI:** 10.1177/23780231241258022

**Published:** 2024-07-29

**Authors:** Mara Getz Sheftel, Noreen Goldman, Anne R. Pebley, Boriana Pratt, Sung S. Park

**Affiliations:** 1 Penn State University, University Park, PA, USA; 2 Princeton University, Princeton, NJ, USA; 3 University of California-Los Angeles, Los Angeles, CA, USA; 4 University of Massachusetts Boston, Boston, MA, USA

**Keywords:** race/ethnicity, occupational segregation, gender, stress, life course

## Abstract

Work, a segregated social context in the United States, may be an important source of differential exposure to stress by race/ethnicity, but existing research does not systematically describe variation in exposure to occupational stress by race/ethnicity. Using work history data from the U.S. Health and Retirement Study and occupational-level measures from the Bureau of Labor Statistics and the Occupational Information Network, the authors document the extent to which the race/ethnicity and gender composition of occupational categories varies by level of occupational strain and how life-course exposure to occupational strain differs by race/ethnicity and gender. Black and Latino workers are overrepresented in high-strain jobs at many ages, compared with other groups. Exposure to job strain across working ages shows more variation in exposure by gender and race/ethnicity groups than static measures. These findings point to potential bias in research using a single, cross-sectional measure of job stress.

Increasing empirical research documents how structural racism plays a central role in shaping differential exposure to stress by race/ethnicity (Brown et al. 2023). This research is focused on how chronic exposure (Turner and Avison 2003) to stress from perceived discrimination (Williams and Mohammed 2013), economic adversity (Brown, Mitchell, and Ailshire 2020; Sternthal, Slopen, and Williams 2011), and social context (Boardman 2004; DeAngelis 2022), drive racial and ethnic health disparities. Work is a critical social context to consider in understanding racialized exposure to stress in the United States because adults spend a considerable portion of their time working (Ahonen et al. 2018). Because of a long legacy of systemic racism affecting educational attainment and discrimination in hiring and advancement, the U.S. labor market is highly segregated by race/ethnicity (King 1992; Tomaskovic-Devey 1993; Weeden, Newhart, and Gelbgiser 2018). Therefore, exposure to occupational stress, or strain (as it is referred to in research on occupations), is likely to vary considerably by race/ethnicity.

Recently, there has been a call to move beyond a static understanding of socially patterned stress, and instead account for the cumulative effects of life-course exposure to such stressors (Hummer 2023). This follows from strong evidence of a “weathering effect” of cumulative exposure to stress resulting in racialized health disparities (Boen 2016; Geronimus 2023; Jackson et al. 2011). The majority of studies on occupational stress use a single job, usually current job or longest held job, to calculate exposure to stress and, in doing so, cannot account for variation in exposure across working ages by race/ethnicity and gender. Additionally, many analyses use samples limited to a specific occupational category (e.g. health care workers) or a specific geographic region (Bennett et al. 2006; Curtis et al. 1997; Hurtado et al. 2012; Landsbergis et al. 2015). Last, investigations of exposure to job stress by race/ethnicity and gender simultaneously are rare even though ample research indicates that multiple identities often intersect to structure opportunity and disadvantage generally (Collins 1994; Crenshaw 1991) and health disparities specifically (Homan, Brown, and King 2021).

In this article we address these limitations by using nationally representative data on the work strain by occupational category and longitudinal work history data to describe differential exposure to job stress by gender and race/ethnicity. To do this, we ask two questions: (1) How do the gender and race/ethnicity composition of occupational categories vary by level of occupational strain? and (2) How does the life-course trajectory of varying levels of occupational strain differ by gender and race/ethnicity? To answer these questions, we analyze how exposure to occupational strain varies by gender and race/ethnicity both at the occupation level and at the individual level over the life course. This analysis makes a critical contribution by being the first to describe how exposure to occupational stress varies across the life course.

## Background

Sociological theory has long considered work an important source of social stratification (Hatt 1950). The high level of occupational segregation in the United States by race/ethnicity (King 1992; Tomaskovic-Devey 1993; Weeden et al. 2018), a downstream product of structural racism, constrains job opportunities for Black and Latino individuals through educational, residential, political, and social exclusion. Discriminatory practices in employment, such as biased hiring processes, wage gaps, and limited career advancement opportunities, disproportionately affect Black and Latino workers (McCall 2001; Quillian and Midtbøen 2021).

**Figure 1.**
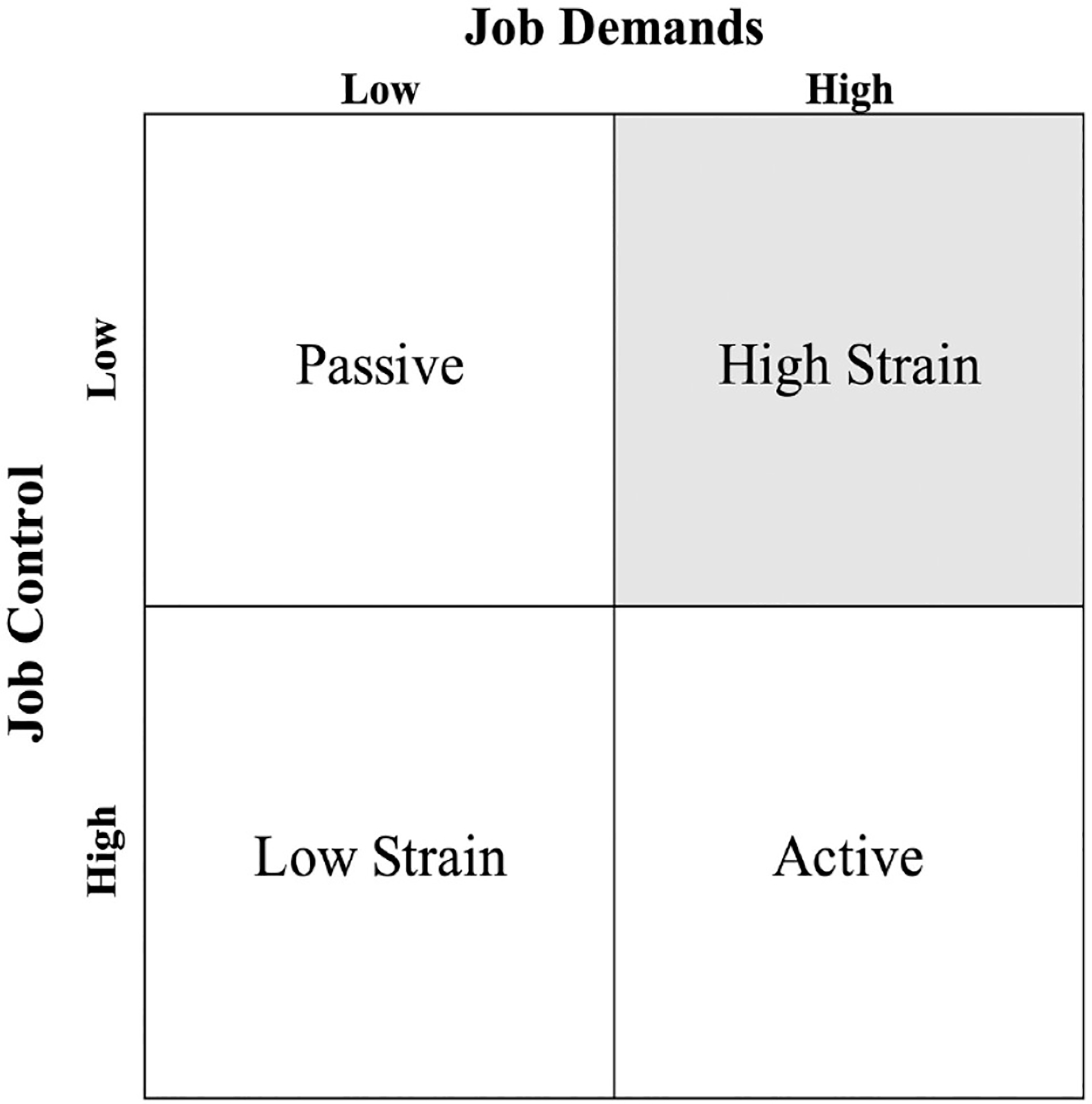
Four job demand-control profiles. *Note*: Adapted from Karasek (1979).

There is evidence that the highly segregated labor market in the United States leads to disproportionate exposure to occupational stress for Black and Latino workers (Bailey et al. 2017; Hurtado et al. 2012; Wadsworth et al. 2007). The most widespread theory of occupational stress, the job demands-control (JDC) model (Karasek 1979; Karasek and Theorell 1990), posits that the level of stress from a job arises from the psychosocial demands required to accomplish that job, in combination with how much control the worker has in meeting those demands. In this context, occupational demand is defined as the level of effort expected to perform a job, and occupational control is defined as the level of decision latitude or individual judgment workers have to complete their job (Andel et al. 2015; Karasek 1979; Karasek and Theorell 1990). The original conceptualization of the JDC model splits both demand and control into two levels, high and low, and offers four combinations of demand-control as illustrated in Figure 1 (adapted from Karasek (1979)). Jobs with both low demand and low control are referred to as passive jobs, and those with both high demand and high control are referred to as active jobs. On the other diagonal, jobs with low demand and high control are low-strain jobs, and those with high demands and low control are high-strain jobs.

Existing research adopting the JDC model of occupational stress seeks to identify patterns of differential exposure to stress by race/ethnicity. Research using a small community-based sample of employees in small manufacturing businesses in eastern Massachusetts found no difference in self-reported occupational strain across race/ethnic groups but men were more likely to report job strain than women (Bennett et al. 2006). Conversely, in a small community-based sample of long-term care workers in Massachusetts, Black workers were more likely to report job strain than White workers (Hurtado et al. 2012). It is unclear if findings about racial/ethnic differences in job strain (and its components) from these applications can be generalized across occupations and localities.

In addition to being focused on a specific geography or occupational sector, existing research documenting differential exposure to occupational strain uses measures of job demand and control from a single job. For example, a study by Raymond, Grzywacz, and Robertson (2022) stands out in the investigators’ use of a nationally representative sample, but they looked only at currently held jobs, finding that Black workers reported less job control than non-Black workers, but no differences in psychological demands. This cross-sectional operationalization of work strain precludes assessing the cumulative and potentially weathering effect of strain (Ferraro and Morton 2018; Geronimus et al. 2006). Cross-sectional measures also assume a constant occupational strain over one’s working life, which is inconsistent with contemporary career mobility (Johnson and Stewart 1993). Despite the importance of life course analyses in sociology, this limitation of research on work strain is unsurprising. Pavalko, Elder, and Clipp (1993) noted that qualitative research on work has long documented longitudinal career patterns, but quantitative work has been slower to consider full work histories, due, in part, to data limitations. To our knowledge, no previous research has systematically documented differential exposure by race/ethnicity to occupational demands and control using a nationally representative sample of workers in civilian occupational sectors, and longitudinal work history data.

The labor market is also segmented by gender and women are more likely than men to work in high-strain jobs (Bonsaksen et al. 2019). Moreover, stress related to work is not only a product of work characteristics themselves, but can also be a function of simultaneously managing work and family responsibilities, a phenomenon that affects women more than men (Hochschild and Machung 2012). Black and Latino women are historically disadvantaged in their exposure to both work and family stress (Roberts 1993) and are disproportionately employed in sectors with high demands but low control or social support like domestic labor and health aides (Dill 2015; Frevert, Culbertson, and Huffman 2015). Therefore, in documenting heterogeneity in exposure to occupational stress, it is important to take an intersectional perspective (Collins 1994; Crenshaw 1991), something we adopt in this analysis.

### Implications of Differential Exposure to Work Strain by Race/Ethnicity and Gender

Why is it important to document differential exposure to occupational stress by race/ethnicity and gender? Chronic stress, including from work, has a known association with adverse health and well-being (Pearlin 1983, 1989; Schieman 2019). If exposure to occupational strain is unequal across race/ethnicity and gender, it may play a role in disparities.

The JDC model posits that high-strain jobs are most likely to be associated with adverse health and well-being (Lerner et al. 1994). Empirical evidence over the past four decades since the model was put forth supports this conclusion (Burgard and Lin 2013). Work in high-strain jobs is associated with a heightened risk for hypertension (Babu et al. 2014; Gilbert-Ouimet et al. 2014; Landsbergis et al. 2015) and cardiovascular disease (Belkic et al. 2004; Slopen et al. 2012) and has an adverse association with cognitive functioning (Agbenyikey et al. 2015; Nilsen et al. 2021), physical function (Nilsen et al. 2019), depression and psychological well-being (Burns, Butterworth, and Anstey 2016). These relationships may partially operate through the positive relationship between high-strain jobs and smoking and high body mass index (Hellerstedt and Jeffery 1997).

High-strain jobs are the primary focus of research using the JDC model to understand implications of occupational strain, but there is also more limited research on the other three JDC categories (Bonsaksen et al. 2019). Active jobs (high demand and high control) and low-strain jobs (low demand and high control) are generally considered more beneficial than high-strain jobs (Lerner et al. 1994). For example, active jobs have been found to be positively associated with cognitive functioning (Andel et al. 2011). However, there is also evidence that high-status jobs, often characterized by high demand and high control, can be detrimental to health (Koltai and Schieman 2015; Schieman and Koltai 2017). Passive jobs (low demand and low control) are less protective than active or low-strain jobs but less detrimental to well-being than high-strain jobs (Agbenyikey et al. 2015; Lerner et al. 1994).

## The Present Study

Based on the JDC conceptualization of work stress, in this analysis we use nationally representative measures of occupational demand and control and longitudinal work history data to describe differential exposure to strain across the life course by race/ethnicity and gender. This lays the ground-work for future research on the consequences of stressful experiences across the life course. To document differences in job strain exposure, we ask the following two questions. First, how do the gender and race/ethnicity compositions of employees in occupational groups vary by type of occupational strain? We answer this question by matching data on the gender, racial, and ethnic composition of specific occupational categories to measures of occupational strain from a nationally representative database, the Occupational Information Network (O*NET). Second, how does the life-course trajectory of engagement in jobs of varying occupational strain differ at the intersection of gender and race/ethnicity? We address this issue using nationally representative work history data to measure exposure to job strain over working ages.

## Methods

### Data Sources

We use three sources of data. To measure occupational strain by occupational category we use data from O*NET. O*NET is collected by the Employment and Training Administration of the U.S. Department of Labor, which randomly samples incumbents (workers) employed in 1,000 occupations from a national sampling frame of establishments. Incumbents answer surveys about occupation-specific tasks, knowledge, education and training, work styles, work activities, and work context. For a minority of occupations, where it is difficult to sample workers, job experts instead of incumbents answer surveys. Data collection for O*NET occurs on a rolling basis, survey responses are aggregated at the occupation level, and summary scores and standard error estimates are released annually so that measures reflect accurate information about occupations as they evolve over time. We use data from O*NET versions 5 (2003), 13 (2008), 18 (2013), and 23 (2018), which are temporally comparable with the Health and Retirement Study (HRS) data that we analyze to understand individual work trajectories (see below). To understand the demographic composition of workers by occupational category, we use estimates of the gender and racial/ethnic makeup of each occupational category produced by the Bureau of Labor Statistics (BLS) using 2018 Current Population Survey data.

To examine the life-course trajectory of work in jobs of varying occupational strain by gender and race/ethnicity, we use data from the HRS, a nationally representative longitudinal panel study of U.S. residents older than 50 years that began in 1992, with waves every two years. HRS is uniquely suited to address our research questions because it includes detailed work histories. We combine data from the restricted access RAND HRS Cross-Year Longitudinal File, which includes detailed occupation codes for jobs held at the time of each interview or most recent job (for those unemployed at time of survey), with retrospective work history data from the restricted 2017 Life History Mail Survey (LHMS), which includes detailed occupation codes for jobs held for at least one year after completing full-time education. This process produces detailed work histories for all HRS respondents in the 2017 LHMS who were employed at any time from completion of full-time education through exit from the labor force or end of observation. Details of the collection of LHMS life history data are described by Smith et al. (2022), and details on the construction of work histories obtained by combining the HRS Core data with LHMS data are described in Park et al. (2022).

In analyzing HRS data, we restrict our analytic sample to U.S.-born White, Black, and Latino respondents in the 2017 LHMS. We exclude respondents from other race/ethnicity groups because of small sample sizes and exclude foreign-born individuals because of concerns about underreporting of work prior to migration to the United States. We include all respondent person-years from 25 until 64 years of age or exit from the sample. Twenty-five is the lower age bound because most respondents have finished full-time education by then, and 64 is the upper age bound so that the work histories end close to the typical age of retirement. Our final analytic sample includes 8,935 respondents with 70,862 person-years.

### Measures

#### Occupational Strain.

We follow previous research using O*NET data to measure the two components of occupational strain: demand and control (Andel et al. 2015; Cifuentes et al. 2007). All O*NET measures are rescaled to range from 0 to 100. For each detailed occupational category, demand is measured by averaging the required level of (1) selective attention, (2) time sharing (shifting between two or more tasks), (3) consequence of error, and (4) importance of being exact or accurate. Likewise, for each detailed occupational category, control is measured by averaging the required level of decision authority and skill discretion on the basis of (1) independence, (2) decision-making freedom, (3) decision-making frequency, (4) impact of decisions on coworkers and company results, and (5) skill discretion.

The JDC model delineates four job profiles (Figure 1), which are a combination of high and low demand and control (Karasek 1979; Karasek and Theorell 1990): (1) passive (low demand, low control), (2) low strain (low demand, high control), (3) high strain (high demand, low control), and (4) active (high demand, high control). Various operationalizations of these four profiles have been proposed (Gómez Ortiz, González, and Segura 2020), but the most widespread method measures the overall range of both demand and control and then uses a median split to categorize occupations into high versus low demand, and high versus low control (Bennett et al. 2006; Landsbergis et al. 2015). Interacting these two measures produces the four JDC profiles. In our data, the national median of occupational demand calculated from all occupations in O*NET version 23 (2018) is 54.3, and the national median of occupational control is 66.9. We apply these cut points to categorize JDC profiles in the HRS work history data.

#### Race/Ethnicity and Gender.

We analyze life-course exposure to occupational demand and control by gender and race/ethnicity using HRS work histories. Race/ethnicity is based on respondents’ self-classification in HRS. For race, HRS requires respondents to self-classify as White, Black, or other and does not have a multiracial category. For ethnicity, HRS requires respondents to self-classify as “not Hispanic” or “Hispanic.” We construct three mutually exclusive race/ethnicity groups: non-Hispanic White (hereafter White), non-Hispanic Black (hereafter Black), and Latino (of any race). Gender is based on respondents’ self-classification as male or female. To investigate occupational strain by race/ethnicity and gender simultaneously, six mutually exclusive groups were constructed: White male, White female, Black male, Black female, Latino male, and Latina female. We limit the analytic sample to those born in the United States because of concerns about the reliability of work history data for foreign-born individuals.

### Analytic Strategy

First, to understand how occupational strain differs by gender and race/ethnicity, we aggregate the rescaled O*NET component scores for demand and control into the 22 major categories^1^ of the 2010 Standard Occupational Classification (SOC) system. We categorize each of the 22 major occupational categories into the four JDC profiles on the basis of their demand and control score (as delineated in the “Measures” section). Table 1 presents the demographic composition of each occupational category (gender, race, and Latino ethnicity^2^), using data published by the BLS and calculated from the 2018 Current Population Survey (Bureau of Labor Statistics 2018b). This descriptive analysis gives us an overall picture of the demographic composition of jobs of varying strain by gender and separately by race/ethnicity, addressing our first research question. We analyze the 22 major categories because BLS suppresses data on the demographic composition of 33 percent of the more detailed “broad group occupations” categories because they have fewer than 50,000 workers and thus do not meet BLS publication criteria. We provide a categorization of each of the 2010 SOC broad group occupations into JDC strain profiles and the gender and race/ethnicity composition of the categories large enough to not be suppressed by the BLS in Appendix A.

Second, to understand the life-course trajectory of job strain by race/ethnicity and gender, we match each job reported in the HRS work history file (Park et al. 2022) with demand and control measures constructed from O*NET. O*NET occupations are classified using the 1,016 O*NET SOC codes, a detailed version of the SOC codes. HRS occupations are classified using U.S. Census Bureau occupation codes. To combine the two datasets, we use a series of cross-walks to convert both HRS-collected job codes and O*NET job codes to SOC codes. Jobs reported in the job history file were matched with measures from the O*NET version temporally closest to when the job information was collected.

**Figure 2.**
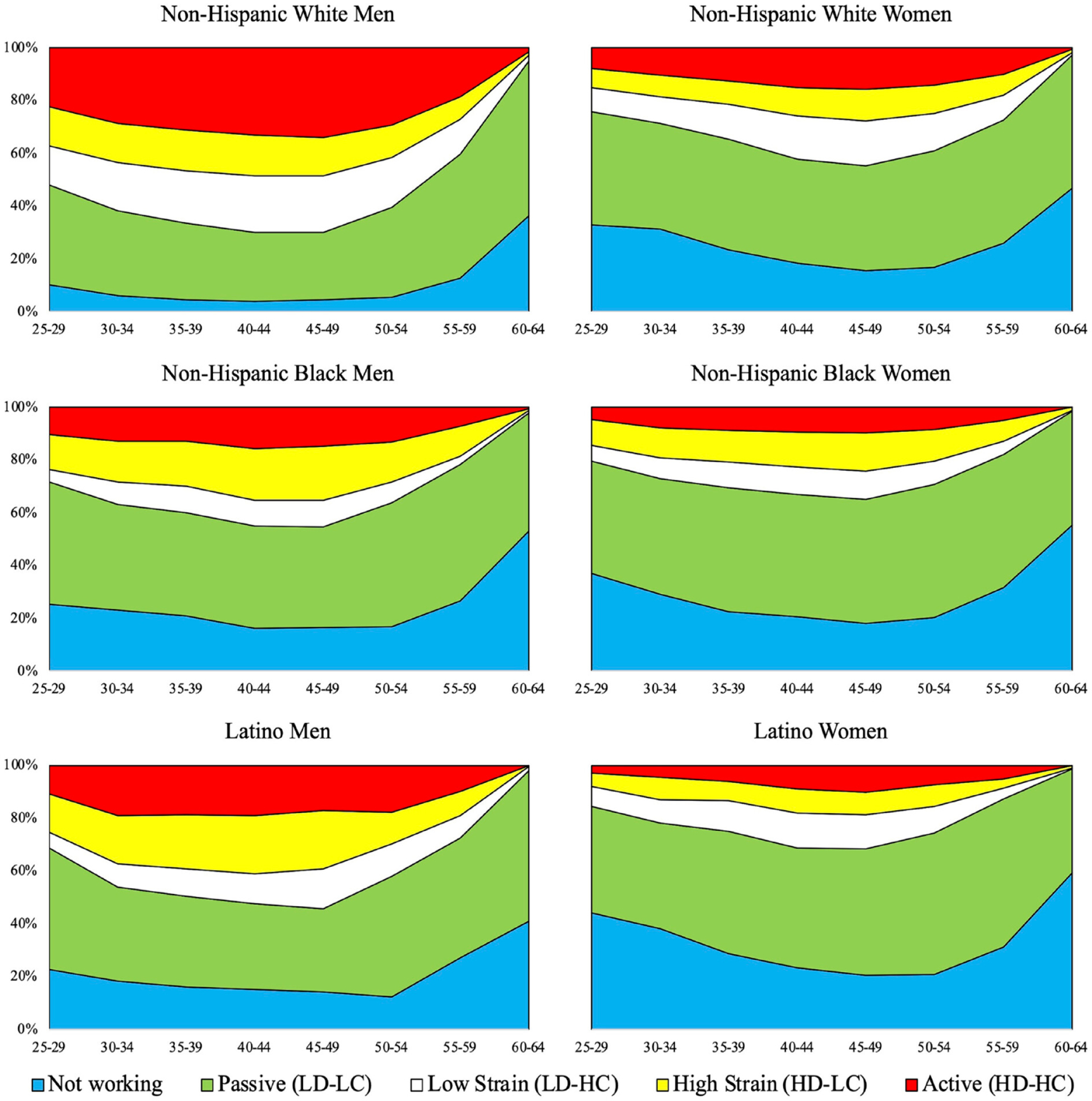
Job strain profiles from 25 to 64 years of age by gender, race, and ethnicity. *Note*: HC = high control, HD = high demand, LC = low control, LD = low demand.

We convert the constructed HRS work history file, which includes time-varying measures of O*NET demand and control for each job held by each individual, to a person-year file for every year from age 25 to age 64 or age of attrition from the survey. For person-years with no employment reported, demand and control are both assigned zero. For person-years when multiple jobs were reported, the average demand and average control scores are taken across all jobs reported for that person-year. Next, we aggregate demand and control for each respondent into five-year age groups, taking the average demand and control score across the five years, and categorize them into one of the four occupational strain profiles (passive, low strain, high strain, and active) or as not working using the same median split as described previously. This results in each respondent having up to eight occupational strain measures (fewer if they exited the survey before age 64). We then calculate the distribution of occupational profiles by age group and gender/race/ethnicity group. These descriptive results are presented in Figure 2 and answer our second research question.

## Results

### JDC Profiles and Occupational Segregation

We first examine how occupational strain and its components, demand and control, differ by gender and race/ethnicity. To facilitate an overall comparison of occupations by JDC profiles, Table 1 categorizes the 22 major occupational categories into JDC strain profiles (passive, low strain, high strain, and active) and presents the gender and race/ethnicity composition of each major category. The top row of Table 1 shows the overall distribution of the employed population in 2018: 46.9 percent of employed people 16 years and older were women, 78.0 percent were White, 12.3 percent were Black, and 17.3 percent were Latino. Darker (red) shading in Table 1 indicates that the corresponding gender or race/ethnicity group is overrepresented in that occupational category compared with the overall distribution of workers, and lighter (yellow) shading indicates that the gender or race/ethnicity group is underrepresented in that category.

Five broad occupational categories are classified as high strain: (1) health care support; (2) construction and extraction; (3) installation, maintenance, and repair; (4) production; and (5) transportation and material moving. Male workers are overrepresented, in some cases to a very large extent, in all of these categories except health care support, in which women make up 87.1 percent of workers. Black workers are overrepresented in three of the five high-strain occupations. For example, in health care support occupations, Black workers make up 26.2 percent of all workers, more than double their representation in the overall employed population. Latino workers are overrepresented in all five of the high-strain occupations. The starkest example is in construction and extraction occupations, in which 37.0 percent of workers were Latino in 2018 compared with 17.3 percent of the overall population distribution. These results indicate that Black and Latino workers, and men in particular, are most likely to hold high-strain jobs.

The three other JDC categories also show evidence of differential exposure to work conditions by race/ethnicity and gender. First, women, Black, and Latino workers are overrepresented in four of the six occupation groups classified as passive. For example, in 2018, office and administrative support employees made up the second largest occupational group in the United States, with more than 17 million individuals older than 16 years employed in these jobs. Of those individuals, 71.6 percent were female workers and 14.4 percent were Black workers. Women and Latino workers are overrepresented in food preparation and serving related occupations and personal care and service occupations. On the other end of the spectrum, active occupations are characterized by a large underrepresentation of women and Black and Latino workers and an overrepresentation of White workers. For example, management occupations, which was the largest occupational group in 2018 with more than 18 million workers, had only 40.0 percent female workers, 7.6 percent Black workers, and 10.3 percent Latino workers.

### JDC across Working Ages by Gender and Race/Ethnicity

The preceding analysis does not provide an examination by gender and race/ethnicity concurrently (because of limitations of BLS data), nor does it offer insight into how exposure to strain may vary across one’s working life. The next part of our analysis, therefore, explores the life-course trajectory of job strain at the intersection of gender and race/ethnicity on the basis of data in the HRS work history file. Figure 2 plots the distribution of JDC profiles, along with a category for those not working, across ages 25–64 by gender and race/ethnicity. Appendix B presents the distributions plotted in Figure 2 and uses an adjusted Wald test to assess statistical significance of race/ethnicity distribution compared with non-Hispanic Whites within JDC profile, age group, and gender. Only differences that are statistically significant (*p* ≤ .05) are mentioned in the following text.

As is evident from the width of the yellow section, representing high-strain jobs, in Figure 2, Latino and Black men have greater overall exposure to high-strain jobs from 40 to 50 years of age^3^ than White men. The age pattern of work in high-strain jobs is similar for Black and Latino men: the percentage increases through the 45- to 49-year age bracket, when it steadily decreases, with fewer than 1 percent of Black and Latino men working in high-strain jobs by ages 60 to 64. Table 2 displays the age group at which each gender-race/ethnicity has the maximum percentage employed in high-strain jobs. The percentages of Black and Latino men in high-strain jobs peak at ages 45 to 49 at 20.4 percent and 22.1 percent, respectively. By contrast, a consistent 14 to 15 percent of White men are employed in high-strain jobs from ages 25 through 49, with a peak of 15.5 percent at ages 40 to 44 before decreasing steadily to about 1 percent by ages 60 to 64.

Within race/ethnicity groups, women are less likely to work in high-strain jobs across working ages than men, consistent with the results from the national-level occupational distribution in Table 1. However, among women, Black women are most likely to work in high-strain jobs through 44 years of age, whereas Latina women have a comparable or lower percentage working in these jobs than White women across most age brackets. These patterns are also visible in Table 2, which shows that the peak percentage of Black women employed in high-strain jobs is 14.3 percent between the ages of 45 and 49, whereas White women peak at 12 percent and Latina women at 9.2 percent (at ages 40–44). Among women, therefore, Black workers have the most exposure to the potentially detrimental characteristics of high-strain jobs, whereas among men, both Latino and Black workers have higher exposure, in multiple age categories, than White men.

Exposure to passive jobs (green) may also be associated with adverse health, and Figure 2 shows evidence of gender and race/ethnicity disparities in exposure to this JDC profile over working ages. For example, across the age range, both Black and Latino men have the highest percent of people working in passive jobs. That is, in each age bracket, the greatest percentage of Black and Latino men work in passive jobs over all other JDC profiles, as well as not working. Among Black men, this percentage ranges from more than one third (38.2 percent) at ages 30 to 34, to more than half (51.5 percent) at ages 55 to 59, with more Black men working in passive jobs than White men across the whole age spectrum. Latino men are more likely than White men to work in passive jobs in the 25- to 29-year and 50- to 54-year age brackets. Women are even more heavily concentrated in passive jobs than men from ages 30 to 54 (59 for Latinos), and Black women are more likely to be employed in passive jobs than White women from ages 30 through 59 (ages 40–59 for Latina women).

Active jobs (red) show the opposite pattern. Consistent with results by occupation in Table 1, White men are more heavily represented in active jobs than Black or Latino men in all age brackets. In each age bracket between 35 and 49 years of age, about one third of White men worked in jobs with high demand and high control. On the other end of the spectrum, the percentage of Black men working in active jobs peaked between ages 40 and 44 at 15.9 percent, about half that of White men working in active jobs. Although the percentage of Latino men in active jobs is higher than that of Black men, it is still considerably lower than that of White men. Across race/ethnicity groups, women are less likely to work in active jobs throughout the age range compared with men. Particularly low percentages of Black and Latina women work in active jobs, peaking for both at 10 percent between ages 45 and 49, compared with White women, who peak at 15.7 percent in the same age range.

## Discussion

This research makes three critical contributions to our understanding of differential exposure to occupational stress by race/ethnicity, and gender. First, by adopting a life-course perspective, and measuring job demand and control across working ages, we show variation in exposure to strain over time. This approach stands out from most empirical research on job strain that measures demand and control at one point in time. Cross-sectional measures of strain can mask important variation over a working life. For example, if job demand and control for the HRS sample had been measured only at 50 to 54 years of age (when many of the HRS respondents first enter the survey), the percentage of men working in high-strain jobs would not vary by race/ethnicity. These findings point to potential bias in research using a single, cross-sectional, measure of job strain. Future research seeking to understand the relationship between occupational stress and older adult well-being should adopt a longitudinal framework.

Second, we measure strain calculated from a nationally representative database (O*NET) and apply cutoffs for high and low demand and control from the distribution of all civilian U.S. occupational categories to a nationally representative sample of older adults. In doing so, we extend previous research using the JDC model of occupational strain, which primarily examines specific occupational categories and/or geographic regions (Bennett et al. 2006; Curtis et al. 1997; Hurtado et al. 2012; Landsbergis et al. 2015).

Third, we use this life-course perspective and nationally representative data to understand differences in exposure to job strain profiles by race/ethnicity and gender. We find that at the national level, Black and Latino workers are overrepresented in high strain and passive occupations and underrepresented in active jobs compared with other groups. These findings point to a potential source of disparities in health and well-being for Black and Latino Americans: Black and Latino workers are more likely to be exposed to adverse psychosocial job characteristics through high-strain jobs (particularly men) and passive jobs (particularly women). This is compounded by the fact that Black and Latino workers also have lower exposure to active jobs, which potentially confer benefits to health and well-being.

Additionally, recent research points to diminished health returns to higher socioeconomic status among darker skinned Black Americans, partly attributable to unfair treatment and perceptions of lower status (DeAngelis, Hargrove, and Hummer 2022). This association could also be important for the occupational strain-health relationship: Black and Latino workers may not derive the same benefit from demand-control profiles that are protective of health as White workers do. Future research, using nationally representative, longitudinal data on exposure to job demand and control should explicitly analyze the contribution of exposure to work strain to health disparities at the intersection of race/ethnicity and gender and consider that the association between health and occupational strain may vary by race and ethnicity.

Our approach is not without limitations. O*NET measures of job characteristics do not include variation within an occupation by race/ethnicity or gender, thereby ignoring structural segregation within an occupation (e.g., systematically different job tasks by gender and race/ethnicity). In fact, Fujishiro and Koessler (2020) compared self-rated job control and O*NET-measured job control by race/ethnicity and found that the association between the two is stronger for White workers compared with other workers. Additionally, our analysis does not address social support in occupational settings. There is evidence that workplace social support may buffer the adverse impact of high-strain jobs, but the buffering effect is not consistent across studies (Cohen and Wills 1985; Haines, Hurlbert, and Zimmer 1991; Jolly, Kong, and Kim 2021). Future longitudinal research building on this analysis should consider differential exposure to workplace social support, in addition to occupational strain. Finally, we do not simultaneously analyze strain from work and family responsibilities, a combination that affects women, particularly Black women. Future research with longitudinal measures of work and family strain should address exposure to these two sources of stress concurrently

Notwithstanding these limitations, this research advances our understanding of differential exposure to occupational stress, by being the first to provide a detailed description of variation in JDC profiles over the life course by race/ethnicity and gender. These results underscore the need for future research that seeks to understand the association between exposure to occupational stress and older adult well-being to take a longitudinal perspective. That is, to assess how disparities in exposure to psychosocial work conditions structure population health disparities, cumulative exposure to occupational stress must be considered.

## Supplementary Material

Appendix B. Distribution of Job-Demand Control Profiles across race/ethnicity, by gender and age

Appendix A: Job Demand-Control and Gender, Race/Ethnicity composition of Broad 2010 SOC Occupational Categories

## Figures and Tables

**Table 1.** Demographic Composition of Occupational Categories by Job Demand-Control Strain Profile.

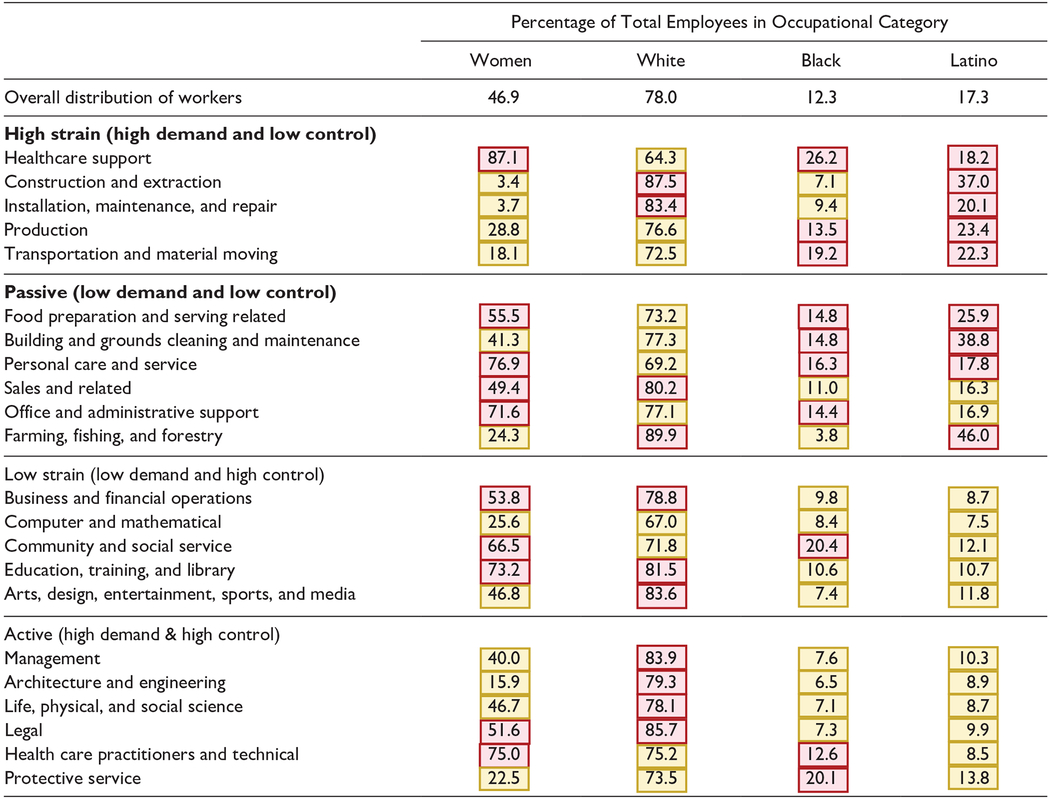

*Note*: Darker (red) shading indicates that the gender or race/ethnicity group is overrepresented in that occupational category compared with the overall distribution of workers. Lighter (yellow) shading indicates that the gender or race/ethnicity group is underrepresented in that occupational category compared with the overall distribution of workers. The job demand-control strain profile was calculated using Occupational Information Network version 23 (2018) measures. The demographic composition of workers 16 years and older in 22 major 2010 Standard Occupational Classification occupational categories is from a Bureau of Labor Statistics (2018a, 2018b) analysis of 2018 Current Population Survey data. Estimates for race/ethnicity groups do not sum to 100 percent, because data are not presented for all races. Those who identify as Latino or Hispanic may be of any race.

**Table 2.** Age of Maximum Percentage Employed in High-Strain Jobs by Gender, Race, and Ethnicity.

	Men		Women	
	Age (y)	%	Age (y)	%
Non-Hispanic White	40–44	15.5	45–49	12.0
Non-Hispanic Black	45–49	20.4	45–49	14.3
Latino	40–44, 45–49	22.1	40–44	9.2

*Note*: Unweighted for full-time or part-time.

## Footnotes

1 O*NET data are not available for military occupations, so the 23rd major occupational category is not included.

2 Those who identify as Latino or Hispanic may be of any race in BLS data (Bureau of Labor Statistics 2018b).

3 In the 55- to 59-year age bracket, a greater percentage of Black men also work in high-strain jobs than White men.
